# Leukocyte-Poor Platelet-Rich Plasma in Foot and Ankle Tendinopathies in Patients With Spondyloarthritis and Rheumatoid Arthritis: A Dual-Center Observational Study

**DOI:** 10.7759/cureus.116952

**Published:** 2026-09-26

**Authors:** Felice Galluccio, Alejandro Fernandez Gibello, Gabriel Camunas Nieves

**Affiliations:** 1 Rheumatology and Pain Management, Fisiotech Lab Studio, Firenze, ITA; 2 Podiatry, Clinica Vitruvio, Madrid, ESP

**Keywords:** enthesitis, plantar fasciitis, platelet-rich plasma, posterior tibial tendon, psoriatic arthritis, regenerative medicine, rheumatoid arthritis, spondyloarthritis, ultrasound

## Abstract

Background: Foot and ankle involvement is common in inflammatory rheumatic diseases. Plantar fasciitis frequently occurs in spondyloarthritis (SpA) and psoriatic arthritis (PsA), whereas tibialis posterior tendon involvement is relevant in rheumatoid arthritis (RA). Evidence for platelet-rich plasma (PRP) in autoimmune disease remains limited.

Objective: This study aimed to evaluate leukocyte-poor PRP (LP-PRP) in SpA/PsA-related plantar fasciitis and RA-related PTT tendinopathy and to assess whether disease activity or structural abnormalities influenced treatment response.

Materials and methods: This dual-center observational study included adults treated with ultrasound-guided LP-PRP. Outcomes included pain (Numeric Rating Scale (NRS)), power Doppler (PWD) activity, effusion, plantar fascia thickness, MRI-detected bone marrow edema (BME), and partial tendon tears. PRP was characterized according to the DEPA classification and administered without exogenous activation. Generalized Estimating Equation models were used to assess changes over time and interactions.

Results: Among 101 patients (68 SpA/PsA; 33 RA), LP-PRP was associated with significant clinical and imaging improvements. In plantar fasciitis, fascia thickness decreased from 0.75 to 0.37 cm and pain from NRS 9 to 1, with resolution of PWD activity. Outcomes were similar between SpA and PsA and were not influenced by disease activity or BME. In RA, pain decreased from 8.36 to 0.48, with resolution of effusion, PWD signal, and partial tears. No disease flares occurred.

Conclusions: Ultrasound-guided LP-PRP was associated with substantial clinical and imaging improvement in inflammatory rheumatic foot and ankle tendinopathies, without observed disease flares. Controlled studies using standardized PRP characterization are warranted.

## Introduction

Foot and ankle involvement is a clinically significant yet often under-recognized manifestation of inflammatory arthritides. Enthesitis is a key pathophysiological process in spondyloarthritis (SpA), including psoriatic arthritis (PsA), with the heel among the most frequently affected sites, particularly at the Achilles tendon and plantar fascia insertions [1]. In axial SpA, plantar fascia enthesitis is characterized by clinical, radiographic, and ultrasound abnormalities, with clear associations between imaging lesions and disease activity and function [2,3]. In PsA, radiographic studies show that calcaneal spurs and plantar periostitis are more frequent than in non-inflammatory conditions and display specific morphological features, reflecting inflammatory enthesopathy at the plantar fascia insertion [4].

Conversely, foot involvement in rheumatoid arthritis (RA) follows a different pathogenic pathway. Rather than enthesitis, which is uncommon, RA frequently leads to tibialis posterior tendon dysfunction and pes planovalgus deformity. Classic observational work has demonstrated that a substantial proportion of RA patients develop clinically evident tibialis posterior tendon dysfunction contributing to planovalgus deformity [5]. Subsequent ultrasound and gait analysis studies have confirmed that tibialis posterior tenosynovitis is common in RA and associated with pes planovalgus, altered kinematics, and functional impairment [6]. In early RA cohorts, tibialis posterior tenosynovitis has emerged as the most frequent ankle and foot lesion detected on ultrasound and a major predictor of functional disability and pain [7]. Together, these data emphasize that plantar fascia enthesitis in SpA/PsA and tibialis posterior tendon involvement in RA represent distinct but equally impactful foot and ankle manifestations.

In recent years, platelet-rich plasma (PRP) has emerged as a regenerative and immunomodulatory technique increasingly applied to chronic tendinopathies. PRP contains a high concentration of platelets and bioactive molecules, including growth factors and cytokines, that can modulate tissue repair, neovascularization, and local inflammatory responses [8,9]. Experimental work has shown that platelet-derived products can enhance mesenchymal cell proliferation, support tenocyte activation, and improve matrix synthesis in tendon models [10-12].

In inflammatory arthritis, in vitro studies show that PRP can suppress rheumatoid fibroblast-like synoviocyte activity and reduce pro-inflammatory mediators, including IL-1β and TNF-α [13,14]. Clinical evidence in RA, mainly from small case series and a randomized study, shows symptomatic improvement and acceptable safety with intra-articular PRP injections [15-17]. In PsA, evidence is limited to isolated case reports of knee synovitis treated with PRP and a report of PsA onset or exacerbation temporally associated with PRP administration [18,19]. Recent narrative and systematic reviews have summarized the emerging role of PRP in rheumatic diseases, while underscoring substantial heterogeneity in products, protocols, and indications, as well as the lack of robust data in patients with active autoimmune diseases [20,21].

Despite the substantial burden of foot and ankle involvement in inflammatory rheumatic diseases, these manifestations remain frequently under-recognized in routine rheumatology practice and comparatively underrepresented in the literature. Plantar fascia enthesopathy in SpA/PsA and tibialis posterior tendon pathology in RA are clinically and pathophysiologically distinct conditions, but both represent relevant examples of persistent local foot pathology that may continue to cause pain and functional impairment despite conservative management and optimized systemic therapy. The present study was therefore designed around this shared clinical gap rather than around a common pathological mechanism. It comprises two parallel disease-specific cohorts treated within the same standardized, ultrasound-guided, non-activated LP-PRP framework and analyzed separately. The objective was to describe the longitudinal clinical and imaging outcomes following LP-PRP treatment in SpA/PsA-related plantar fascia involvement and RA-related tibialis posterior tendon involvement and, within each cohort, to explore whether systemic disease activity and relevant imaging abnormalities were associated with treatment response.

## Materials and methods

This dual-center observational study was a retrospective secondary analysis of prospectively recorded clinical data obtained during routine care at two expert centers specializing in foot and ankle pathology and inflammatory rheumatic diseases. The study comprised two parallel disease-specific cohorts: patients with SpA or PsA and plantar fasciitis and patients with RA and tibialis posterior tendon involvement. The two cohorts were analyzed separately and were not pooled for comparative analysis.

Although clinical data were recorded prospectively, this study was not prospectively designed. No patients were enrolled according to a research protocol, and no study-specific visits, imaging examinations, interventions, or assessments were introduced. Baseline and follow-up evaluations at one, two, and three months were part of the standardized clinical practice of the participating centers. The existing data were subsequently anonymized and analyzed retrospectively.

The study was coordinated in Italy within an established clinical collaboration between two centers, one in Italy and one in Spain. Investigators involved in the study conduct clinical activity across both centers, which use the same standardized clinical treatment and follow-up pathway.

All participants had provided written informed consent for treatment and for the use of their anonymized clinical information for research purposes. In accordance with the applicable regulatory framework for retrospective non-pharmacological analyses of anonymized routinely collected clinical data, formal prior ethics committee approval was not obtained, and no ethics committee approval number was generated.

Consecutive clinical registry records from January 2022 through March 2026 were screened using two disease- and target-specific combinations: RA with tibialis posterior tendon involvement and PsA/SpA with plantar fasciitis. The initial registry search identified 152 potentially eligible patients: 48 with RA and tibialis posterior tendon involvement and 104 with PsA/SpA and plantar fasciitis.

Eligibility was subsequently assessed based on the predefined inclusion and exclusion criteria and the availability of complete follow-up data. Fifty-one patients were excluded. Of these, eight (15.7%) had incomplete follow-up data, 11 (21.6%) had previously received an injection at the target site, and 32 (62.7%) had not consented to research use of their clinical data. The final analytic population therefore comprised 101 patients: 33 in the RA/tibialis posterior tendon cohort and 68 in the PsA/SpA plantar fascia cohort. All patients included in the final analysis had complete data at the scheduled assessment points.

Eligible patients were adults (≥18 years) with a confirmed diagnosis of RA, PsA, or SpA according to established classification criteria: the 2010 American College of Rheumatology/European Alliance of Associations for Rheumatology (ACR/EULAR) criteria for RA [22], the Classification Criteria for Psoriatic Arthritis (CASPAR) criteria for PsA [23], and the Assessment of Spondyloarthritis International Society (ASAS) criteria for axial or peripheral SpA [24].

Patients were included if they had chronic symptomatic tibialis posterior tendinopathy or tenosynovitis in the RA cohort or plantar fasciitis in the PsA/SpA cohort, refractory to conservative management including physiotherapy, nonsteroidal anti-inflammatory drugs (NSAIDs), orthoses, and optimized systemic disease-modifying antirheumatic drug (DMARD) therapy. Exclusion criteria included active infection, coagulopathy, pregnancy, recent corticosteroid injection or other injection therapy at the target tendon or fascia, and previous surgery at the target site.

An experienced rheumatologist or podiatrist performed ultrasound evaluations using GE ultrasound systems (GE LOGIQ Fortis and GE S7 Expert, GE HealthCare, Chicago, IL, USA) equipped with high-frequency linear probes (ML6-15, L8-18, and L6-24). In the RA cohort, ultrasound assessment of the tibialis posterior tendon included tendon-sheath effusion, power Doppler (PWD) activity, and the presence or absence of a partial tendon tear. Effusion and PWD activity were recorded using semiquantitative 0-3 scales, with grade 0 indicating absence and grades 1-3 indicating increasing severity. Partial tendon tear was recorded as a binary variable (present/absent). Pain intensity was measured using the 11-point Numeric Rating Scale (NRS: 0-10) [25].

In the PsA/SpA cohort, plantar fascia assessment included fascial thickness and PWD activity. Fascial thickness was recorded in centimeters. PWD was assessed using a semiquantitative 0-3 scale, with positivity defined as any detectable Doppler signal (grade >0). Pain was assessed using the NRS. Magnetic resonance imaging (MRI) was used to record the presence or absence of bone marrow edema (BME), which was analyzed as a binary imaging variable. BME was assessed at baseline and at the three-month evaluation.

No post hoc blinded central rereading of stored ultrasound images was performed, and no formal interobserver or intraobserver reliability analysis was part of the study design. The analysis was based on the imaging findings recorded during the clinical assessments.

Because this was a retrospective secondary analysis, no research-specific primary or secondary endpoint was prospectively designated. For this analysis, longitudinal outcomes were defined based on the clinical and imaging variables available for each disease-specific cohort. In the PsA/SpA plantar fascia cohort, these included NRS pain, plantar fascia thickness, and PWD activity. MRI-detected BME was evaluated as an imaging variable and as a baseline factor potentially associated with subsequent clinical and ultrasound trajectories. In the RA tibialis posterior tendon cohort, outcomes included NRS pain, tendon-sheath effusion, PWD activity, and partial tendon tears.

PRP preparation followed the DEPA framework described by Magalon et al. [26]. LP-PRP was prepared to achieve a platelet concentration of approximately 1 × 10⁶/µL ± 20% in a final volume of 3-5 mL. No exogenous activator was used. All injections were performed under continuous ultrasound guidance using sterile technique and single-use sterile materials.

Platelet quantification was performed manually using a Bürker hemocytometer after dilution in Türk’s solution. The diluted PRP was loaded into the chamber by capillarity and allowed to settle before microscopic platelet counting within the designated grid areas. Platelet concentration was calculated according to the dilution factor and chamber volume. Manual counting was used for quality control and verification of platelet concentration.

For plantar fascia procedures, a tibial nerve block was performed before PRP administration using 4 mL of 1% lidocaine, following the ultrasound-guided technique described by Fernández-Gibello et al. [27]. PRP was subsequently delivered using a 25G × 0.50 × 40 mm or 27G × 0.40 × 40 mm needle, selected according to tissue depth and anatomical requirements. In the RA cohort, PRP was injected into the tibialis posterior tendon sheath and the involved tendon region under ultrasound guidance. In the PsA/SpA cohort, PRP was injected into the affected areas of the plantar fascia.

During follow-up, patients were instructed to avoid anti-inflammatory medications, including NSAIDs, COX-2 inhibitors, and corticosteroids. Non-anti-inflammatory analgesics such as acetaminophen or opioids, together with cryotherapy and orthotic devices, were permitted when clinically required. Systemic treatment for RA, PsA, or SpA, including conventional DMARDs, biologic agents, and targeted therapies, remained unchanged during the observational period.

Exploratory subgroup analyses examined clinically relevant baseline factors within each disease-specific cohort. In the PsA/SpA cohort, longitudinal pain and plantar fascia thickness were evaluated according to disease type (AS versus PsA), baseline systemic disease activity, and baseline MRI-detected BME. PWD and longitudinal MRI findings were summarized descriptively. In the RA cohort, the association between baseline disease activity and longitudinal pain was examined inferentially, whereas changes in effusion, PWD activity, and partial tendon tears were analyzed descriptively.

Quantitative variables were summarized using mean and standard deviation, whereas categorical variables were reported as absolute and relative frequencies (n (%)). Longitudinal continuous outcomes were analyzed using Generalized Estimating Equation (GEE) models with an exchangeable working correlation structure and empirical standard errors to account for repeated measurements within subjects. Statistical significance of model parameter estimates was assessed using Wald z-tests. Pairwise comparisons of least-squares means across time points were adjusted for multiple comparisons using the Tukey-Kramer method. Relevant subgroup variables and time interactions were incorporated into the corresponding GEE models, where inferential subgroup analyses were performed. Statistical significance was set at a two-sided p-value <0.05.

No longitudinal outcome data were missing at the scheduled assessment points among patients included in the final analytic population; therefore, no imputation was required. When an empirical standard error was zero because a within-subject contrast showed no variability, a finite Wald z statistic could not be estimated and was therefore not reported.

## Results

The analysis included 101 patients: 68 with plantar fasciitis associated with PsA or SpA and 33 with tibialis posterior tendon involvement associated with RA. Table 1 summarizes the baseline demographic and clinical characteristics of the study population.

**Table 1:** Baseline demographic, clinical, and imaging characteristics of the study cohorts Values are reported as mean ± SD for continuous variables and as n (%) for categorical variables. PWD positivity was defined as the presence of any detectable PWD signal (grade >0). AS: ankylosing spondylitis, PsA: psoriatic arthritis, RA: rheumatoid arthritis, NRS: Numeric Rating Scale, MRI: magnetic resonance imaging, PWD: power Doppler, SD: standard deviation, BME: bone marrow edema. The dash (-) indicates not applicable.

Variable	Plantar fascia SpA/PsA (n = 68)	Tibialis posterior tendon RA (n = 33)
Age, mean ± SD (years)	49.2 ± 17.1	48.0 ± 19.3
Female, n (%)	40 (58.8%)	23 (69.7%)
Male, n (%)	28 (41.2%)	10 (30.3%)
AS, n (%)	25 (36.8%)	-
PsA, n (%)	43 (63.2%)	-
RA, n (%)	-	33 (100%)
Active disease at baseline, n (%)	29 (42.6%)	17 (51.5%)
Pain NRS (0-10), mean ± SD	9.01 ± 0.82	8.36 ± 0.93
Fascial thickness (cm), mean ± SD	0.75 ± 0.14	-
BME on MRI, n (%)	21 (30.9%)	-
PWD positive, n (%)	23 (33.8%)	33 (100%)
Effusion, n (%)	-	33 (100%)
Partial tendon tear, n (%)	-	11 (33.3%)

Plantar fascia cohort

The plantar fascia cohort included 68 patients: 43 with PsA and 25 with AS. Mean fascial thickness progressively decreased from 0.745 ± 0.14 cm at baseline to 0.674 ± 0.14 cm at one month, 0.455 ± 0.09 cm at two months, and 0.372 ± 0.04 cm at three months. Compared with baseline, the reduction was significant at one month (Wald z = -3.08, p = 0.002), two months (Wald z = -14.63, p < 0.001), and three months (Wald z = -21.33, p < 0.001). Figure 1 illustrates the progressive reduction in plantar fascia thickness over time and across the analyzed subgroups.

**Figure 1:**
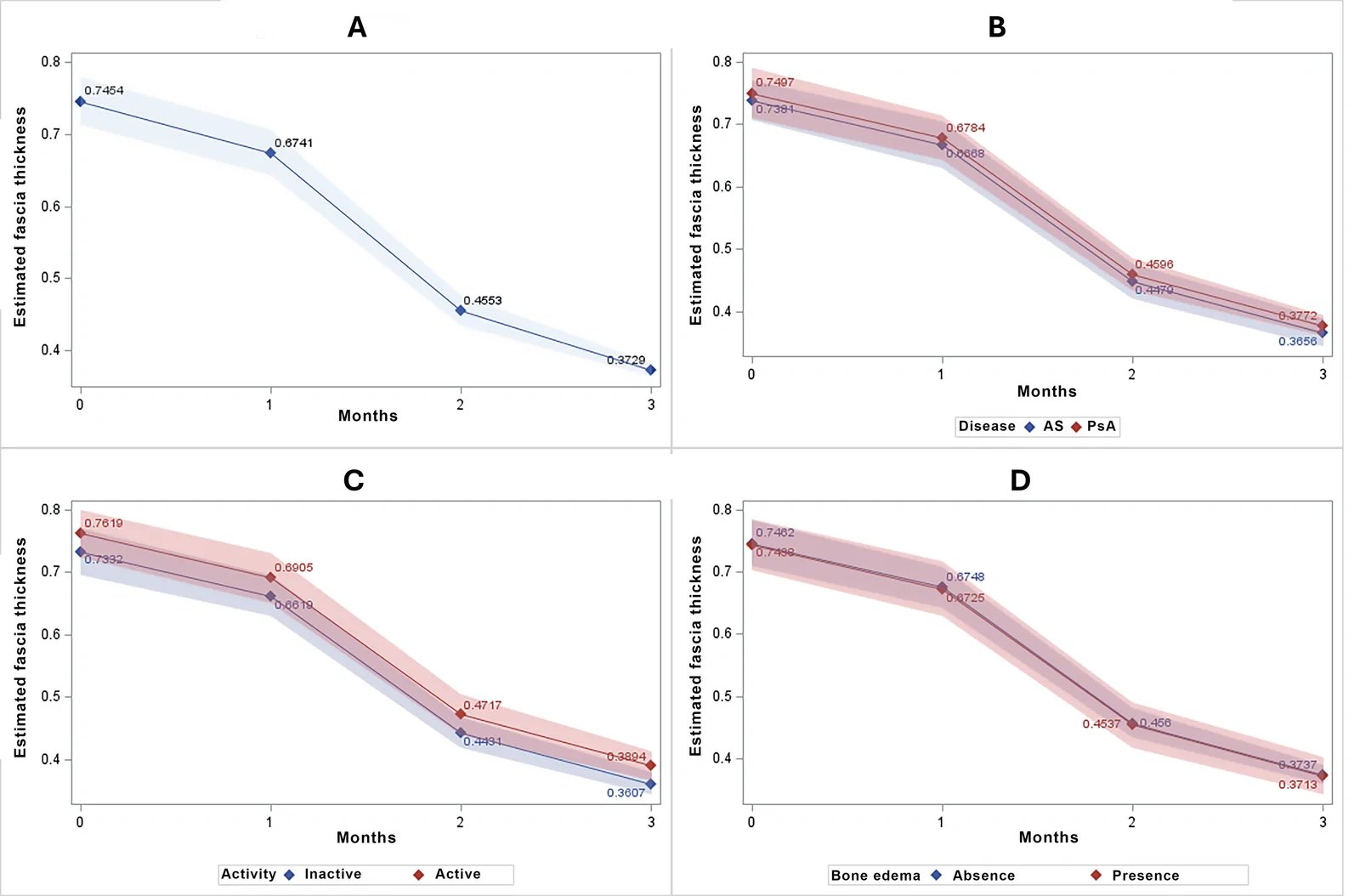
Changes in plantar fascia thickness over time (A) Overall temporal evolution of plantar fascia thickness following LP-PRP treatment. (B) Changes in plantar fascia thickness stratified by disease type (AS vs PsA). (C) Changes stratified by baseline disease activity (active vs inactive disease). (D) Changes stratified by the presence or absence of baseline BME on MRI. Fascial thickness progressively decreased throughout the three-month follow-up. No significant differences were observed according to disease type (Wald z = -0.66, p = 0.508), baseline disease activity (Wald z = 1.54, p = 0.123), or baseline BME (Wald z = -0.11, p = 0.910). AS: ankylosing spondylitis, PsA: psoriatic arthritis, LP-PRP: leukocyte-poor platelet-rich plasma, MRI: magnetic resonance imaging, BME: bone marrow edema

Pain improved in parallel and substantially, with mean NRS scores decreasing from 9.01 ± 0.82 at baseline to 7.01 ± 0.82 at one month, 4.01 ± 0.82 at two months, and 1.01 ± 0.82 at three months. The identical standard deviations across assessment points reflect identical marginal score-frequency distributions shifted toward lower NRS values over time. Compared with baseline, pain decreased significantly at one month (Wald z = −96.17, p < 0.001) and two months (Wald z = −50.13, p < 0.001). At three months, all 68 patients showed an 8-point reduction from baseline, resulting in zero variability in the within-subject change from T0 to T3 and an empirical standard error of zero. Therefore, a finite Wald z statistic and corresponding Wald-based p-value were not estimable for this contrast. Figure 2 shows the evolution of pain scores over the three-month follow-up.

**Figure 2:**
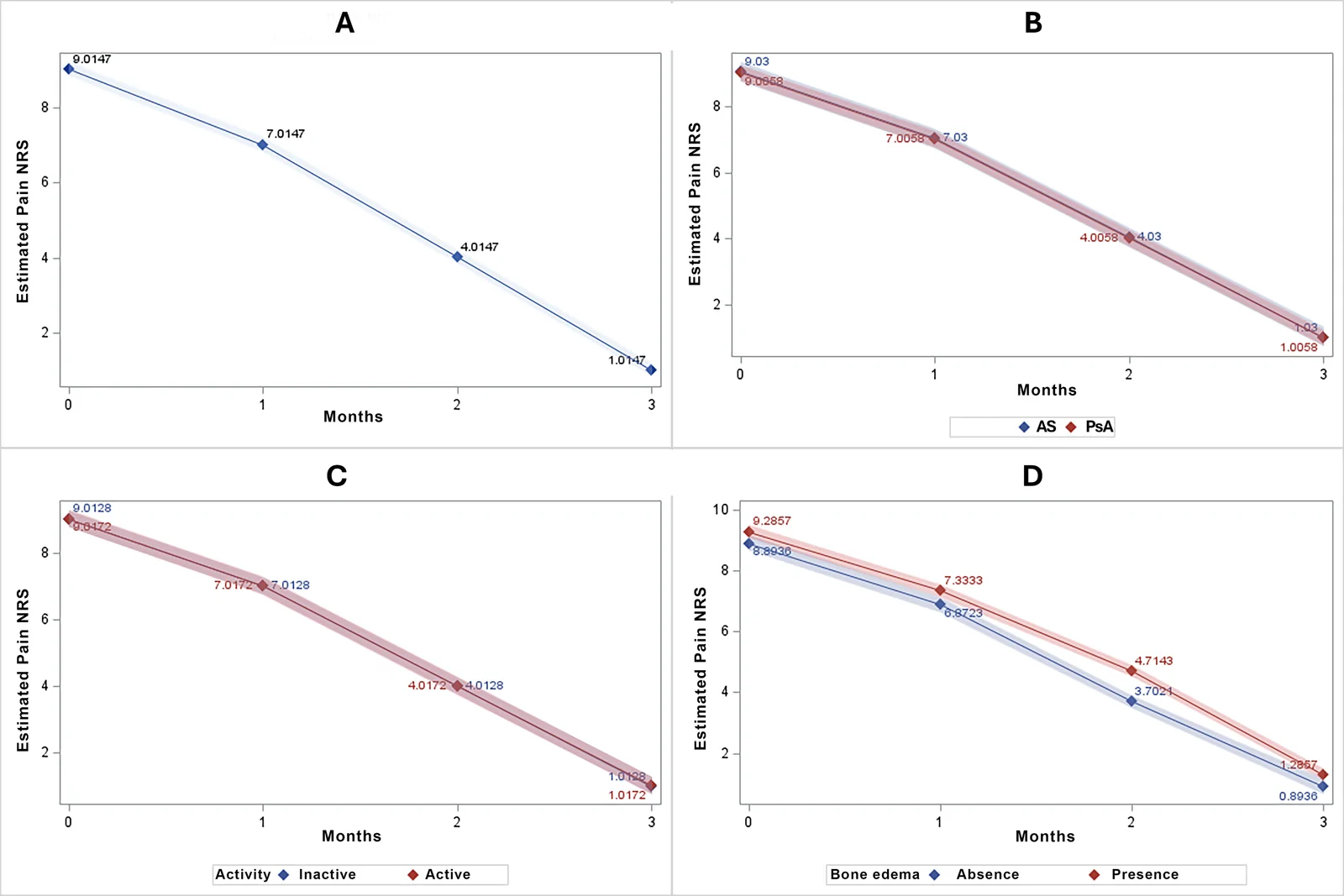
Evolution of pain (NRS) in plantar fasciitis (A) Overall temporal evolution of NRS pain scores following LP-PRP treatment. (B) Evolution of pain stratified by disease type (AS vs PsA). (C) Evolution of pain stratified by baseline disease activity (active vs inactive disease). (D) Evolution of pain stratified by the presence or absence of BME on baseline MRI. Pain decreased markedly throughout the three-month follow-up. Pain scores did not differ significantly by disease type (Wald z = 0.13, p = 0.896) or baseline disease activity (Wald z = 0.02, p = 0.980). In contrast, baseline BME was associated with higher pain scores overall (Wald z = 2.38, p = 0.017), with a significant BME-by-time effect at two months (Wald z = 2.79, p = 0.005). NRS: Numeric Rating Scale, AS: ankylosing spondylitis, PsA: psoriatic arthritis, BME: bone marrow edema, MRI: magnetic resonance imaging, LP-PRP: leukocyte-poor platelet-rich plasma

PWD activity also progressively decreased. At baseline, PWD was positive in 23 (33.8%) patients, whereas 45 (66.2%) had no detectable Doppler signal. At one month, PWD remained detectable in 14 (20.6%) patients and was absent in 54 (79.4%). At both two and three months, PWD activity was absent in 68 (100%) patients. MRI-detected BME was present in 21 (30.9%) patients at baseline and was absent in 68 (100%) patients at the three-month assessment.

Disease activity was not significantly associated with fascial thickness (Wald z = 1.54, p = 0.123) or pain scores (Wald z = 0.02, p = 0.980). Similarly, no significant differences were observed between AS and PsA for fascial thickness (Wald z = -0.66, p = 0.508) or pain (Wald z = 0.13, p = 0.896). Baseline BME was not significantly associated with fascial thickness (Wald z = -0.11, p = 0.910). However, in the pain model, BME was associated with higher pain scores overall (Wald z = 2.38, p = 0.017), with a significant BME-by-time effect at two months (Wald z = 2.79, p = 0.005). Table 2 summarizes the longitudinal clinical and imaging outcomes of the plantar fascia cohort.

**Table 2:** Plantar fascia outcomes (SpA/PsA) Continuous variables are reported as mean ± SD, and categorical variables as n (%). Longitudinal continuous outcomes were analyzed using GEE models with empirical standard errors, and model parameter estimates were assessed using Wald z-tests. PWD and longitudinal MRI findings are presented descriptively. For the T3-versus-T0 pain contrast, all 68 patients showed an 8-point reduction from baseline, resulting in zero within-subject variability and, consequently, an empirical standard error of zero. Therefore, a finite Wald z statistic and corresponding Wald-based p-value could not be estimated. NRS: Numeric Rating Scale, PWD: power Doppler, BME: bone marrow edema, MRI: magnetic resonance imaging, AS: ankylosing spondylitis, PsA: psoriatic arthritis, NE: not estimable, SD: standard deviation, GEE: Generalized Estimating Equation

Outcome	Analysis/comparison	Baseline	1 month	2 months	3 months	Wald z	p-value
Fascial thickness (cm)	Mean ± SD	0.745 ± 0.14	0.674 ± 0.14	0.455 ± 0.09	0.372 ± 0.04	-	-
Fascial thickness	T1 vs T0	-	-	-	-	−3.08	0.002
Fascial thickness	T2 vs T0	-	-	-	-	−14.63	<0.001
Fascial thickness	T3 vs T0	-	-	-	-	−21.33	<0.001
Pain NRS (0–10)	Mean ± SD	9.01 ± 0.82	7.01 ± 0.82	4.01 ± 0.82	1.01 ± 0.82	-	-
Pain NRS	T1 vs T0	-	-	-	-	−96.17	<0.001
Pain NRS	T2 vs T0	-	-	-	-	−50.13	<0.001
Pain NRS	T3 vs T0	-	-	-	-	NE*	NE*
PWD positive, n (%)	Descriptive	23 (33.8%)	14 (20.6%)	0 (0%)	0 (0%)	-	-
BME, n (%)	Descriptive	21 (30.9%)	-	-	0 (0%)	-	-
Baseline BME	Effect on fascial thickness	-	-	-	-	−0.11	0.91
Baseline BME	Effect on pain	-	-	-	-	2.38	0.017
Baseline BME × time	Effect at T2	-	-	-	-	2.79	0.005
Disease activity	Effect on fascial thickness	-	-	-	-	1.54	0.123
Disease activity	Effect on pain	-	-	-	-	0.02	0.98
AS vs PsA	Effect on fascial thickness	-	-	-	-	−0.66	0.508
AS vs PsA	Effect on pain	-	-	-	-	0.13	0.896

Tibialis posterior tendon cohort

The tibialis posterior tendon cohort included 33 patients with RA. Mean pain NRS decreased from 8.36 ± 0.93 at baseline to 4.97 ± 0.88 at one month, 2.30 ± 0.98 at two months, and 0.48 ± 0.62 at three months. Compared with baseline, pain decreased significantly at one month (Wald z = -15.86, p < 0.001), two months (Wald z = -28.32, p < 0.001), and three months (Wald z = -40.35, p < 0.001).

Ultrasound abnormalities improved progressively during follow-up. At baseline, effusion was grade 2 in 11 (33.3%) patients and grade 3 in 22 (66.7%). At one month, effusion was grade 1 in 11 (33.3%), grade 2 in 13 (39.4%), and grade 3 in nine (27.3%) patients. By two months, effusion was absent in 21 (63.6%) patients and limited to grade 1 in 12 (36.4%); at three months, effusion was absent in all 33 (100%) patients.

PWD improved similarly. At baseline, PWD was grade 2 in 11 (33.3%) patients and grade 3 in 22 (66.7%). At one month, grade 1, grade 2, and grade 3 PWD activity were each observed in 11 (33.3%) patients. At two months, PWD was absent in 25 (75.8%) patients and persisted as grade 1 in eight (24.2%). At three months, PWD activity was absent in all 33 (100%) patients.

Partial tendon tears were present in 11 (33.3%) patients at baseline and remained detectable in 11 (33.3%) at one month. At two months, partial tears persisted in nine (27.3%) patients, whereas 24 (72.7%) no longer showed evidence of tearing. At three months, no partial tendon tears were detected in any of the 33 patients. Baseline RA disease activity was not significantly associated with pain scores in the GEE model (Wald z = 1.12, p = 0.261). Table 3 summarizes the longitudinal clinical and ultrasound outcomes for the tibialis posterior tendon cohort.

**Table 3:** Tibialis posterior tendon outcomes in patients with RA Continuous variables are reported as mean ± SD, and categorical variables as n (%). Longitudinal changes in pain were analyzed using a GEE model with empirical standard errors, and model parameter estimates were assessed using Wald z-tests. Effusion, PWD activity, and partial tendon tears are presented descriptively because no corresponding inferential longitudinal test was performed for these outcomes. NRS: Numeric Rating Scale, PWD: power Doppler, RA: rheumatoid arthritis, SD: standard deviation, GEE: Generalized Estimating Equation

Outcome	Analysis/category	Baseline	1 month	2 months	3 months	Wald z	p-value
Pain NRS (0-10)	Mean ± SD	8.36 ± 0.93	4.97 ± 0.88	2.30 ± 0.98	0.48 ± 0.62	-	-
Pain NRS	T1 vs T0	-	-	-	-	−15.86	<0.001
Pain NRS	T2 vs T0	-	-	-	-	−28.32	<0.001
Pain NRS	T3 vs T0	-	-	-	-	−40.35	<0.001
Effusion	Grade 1, n (%)	0 (0%)	11 (33.3%)	12 (36.4%)	0 (0%)	-	-
Effusion	Grade 2, n (%)	11 (33.3%)	13 (39.4%)	0 (0%)	0 (0%)	-	-
Effusion	Grade 3, n (%)	22 (66.7%)	9 (27.3%)	0 (0%)	0 (0%)	-	-
Effusion	Absent, n (%)	0 (0%)	0 (0%)	21 (63.6%)	33 (100%)	-	-
PWD	Grade 1, n (%)	0 (0%)	11 (33.3%)	8 (24.2%)	0 (0%)	-	-
PWD	Grade 2, n (%)	11 (33.3%)	11 (33.3%)	0 (0%)	0 (0%)	-	-
PWD	Grade 3, n (%)	22 (66.7%)	11 (33.3%)	0 (0%)	0 (0%)	-	-
PWD	Absent, n (%)	0 (0%)	0 (0%)	25 (75.8%)	33 (100%)	-	-
Partial tendon tear	Present, n (%)	11 (33.3%)	11 (33.3%)	9 (27.3%)	0 (0%)	-	-
Partial tendon tear	Absent, n (%)	22 (66.7%)	22 (66.7%)	24 (72.7%)	33 (100%)	-	-
Disease activity	Effect on pain	-	-	-	-	1.12	0.261

The marked reduction in pain over the three-month follow-up was observed in both the overall RA cohort and after stratification by baseline disease activity. Figure 3 shows the overall pain trajectory and the corresponding trajectories stratified by baseline disease activity.

**Figure 3:**
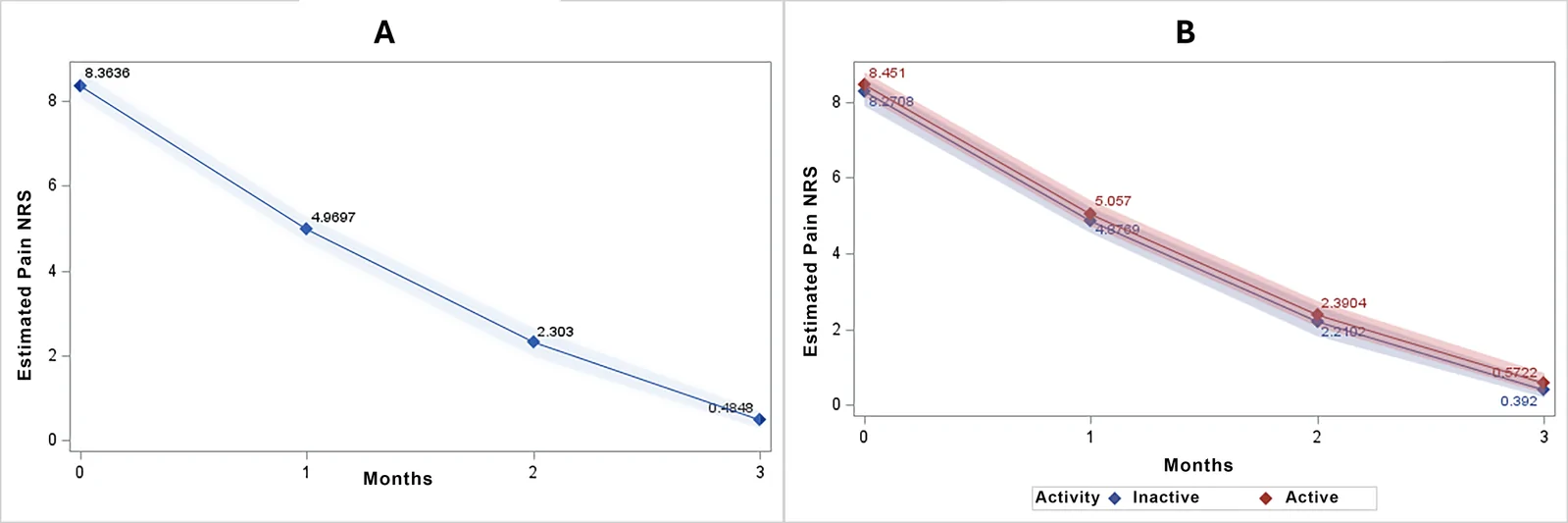
Evolution of pain in RA patients with tibialis posterior tendon involvement (A) Overall temporal evolution of NRS pain scores in patients with RA and tibialis posterior tendinopathy/tenosynovitis treated with LP-PRP. Pain decreased progressively from baseline through the three-month follow-up. (B) Evolution of NRS pain scores stratified by baseline RA disease activity. Patients with active and inactive disease showed similar pain trajectories, and baseline disease activity was not significantly associated with pain scores in the GEE model (Wald z = 1.12, p = 0.261). NRS: Numeric Rating Scale, RA: rheumatoid arthritis, LP-PRP: leukocyte-poor platelet-rich plasma, GEE: Generalized Estimating Equation

## Discussion

This dual-center observational experience suggests that standardized LP-PRP treatment is associated with rapid and clinically meaningful improvements in pain and imaging abnormalities in two distinct but clinically relevant foot and ankle manifestations of inflammatory arthritis: plantar fascia enthesopathy in SpA/PsA and tibialis posterior tendon involvement in RA.

Our findings are consistent with the evolving understanding of plantar fascia involvement in SpA. Several studies have shown that plantar fascia enthesitis is common in axial SpA and can be detected by clinical, radiographic, and ultrasound assessment, with good correlation with disease activity and functional indices [2,3]. Radiographic data in PsA demonstrate that calcaneal spurs and plantar periostitis are more prevalent and morphologically distinct from degenerative spurs, underscoring the inflammatory nature of heel enthesopathy in this disease [4]. The observed clinical and imaging changes in our cohort suggest that local regenerative approaches warrant further investigation as potential adjuncts to systemic therapy in patients with persistent local symptoms despite otherwise optimized treatment.

Similarly, our results align with the recognized role of tibialis posterior tendon pathology in RA. Early work by Michelson et al. showed that a significant proportion of patients with RA develop tibialis posterior tendon dysfunction with planovalgus deformity [5]. More recent studies using ultrasound, gait analysis, and electromyography have confirmed that tibialis posterior tenosynovitis and pes planovalgus are common, are associated with altered foot mechanics, and contribute substantially to disability [6,7]. This is further supported by a recent clinical practice guideline for foot and ankle management in RA, which emphasizes targeted assessment and localized therapeutic strategies to address tendon dysfunction and structural deformities in this patient population [28].

The marked reduction in pain together with the progressive improvement in ultrasound abnormalities observed in our RA cohort suggests that treatment directed at local tendon pathology may provide clinically relevant benefit even in the context of systemic inflammatory disease. Notably, baseline RA disease activity was not significantly associated with pain scores, suggesting that local symptomatic improvement may occur regardless of overall systemic disease activity. However, because inferential subgroup analyses of effusion, PWD activity, and partial tendon tears were not performed, no conclusions regarding the influence of systemic disease activity on these individual ultrasound outcomes can be drawn. An important aspect of this study was the disease-specific and anatomical selection of patients. The cohorts did not include patients with nonspecific foot or ankle pain simply based on an underlying rheumatic diagnosis. The symptomatic tendon or enthesis was identified through specialist clinical and ultrasound assessment, with attention to alternative structural and biomechanical causes of symptoms. This distinction is particularly relevant in weight-bearing tendons and entheses, where mechanical loading and inflammatory activity are biologically interrelated. Experimental models of SpA show that mechanical unloading markedly suppresses Achilles enthesitis, whereas restoring mechanical strain reactivates mechanotransduction pathways; subsequent work has also shown that mechanical strain contributes to the site-specific localization of inflammation and tissue damage [29,30].

From a pathophysiologic standpoint, these clinical observations are supported by in vitro and translational data. PRP contains a broad array of growth factors and cytokines involved in tissue repair, angiogenesis, and inflammation modulation [8,9]. These findings are consistent with a recent review summarizing the regenerative and immunomodulatory actions of PRP across several rheumatic diseases and supporting its potential role as an adjunctive therapy in inflammatory and degenerative musculoskeletal conditions [31].

Experimental models have demonstrated that PRP and platelet releasate can enhance mesenchymal cell proliferation, support tenocyte activity, and optimize extracellular matrix production [10-12]. In RA-specific models, PRP has also been shown to modulate pro-inflammatory pathways and reduce the pathogenic activity of fibroblast-like synoviocytes, including modulation of pathways involving galectins and TGF-β signaling [13,14].

Clinical evidence in RA remains limited but supports the potential benefit of intra-articular PRP. Case series and randomized trials have reported pain relief and functional improvement in patients with RA receiving PRP injections, with acceptable safety profiles [15-17]. These observations align with a 2021 systematic review reporting encouraging but still preliminary evidence for PRP in RA, while emphasizing the heterogeneity and limited methodological quality of the available studies [32].

In PsA, evidence remains particularly scarce. Published data include a case report of knee synovitis treated with PRP with apparent clinical improvement [18], as well as an isolated report of PsA developing or flaring after PRP administration [19]. These observations raise theoretical concerns about using platelet-derived biologic products in inflammatory disease, although larger clinical series have not substantiated them. In the present cohorts, no disease flares were observed during follow-up, including among patients with active systemic disease, which provides preliminary reassurance regarding short-term tolerability.

An additional finding of interest concerned BME in the plantar fascia cohort. Baseline BME was not significantly associated with fascial thickness, suggesting that the structural response of the plantar fascia was similar regardless of MRI-detected bone involvement. However, the pain model showed higher pain scores overall among patients with BME and a significant BME-by-time effect at two months. This suggests that BME may influence the early symptomatic trajectory without necessarily affecting the structural response of the fascia. Given the relatively small subgroup size and the exploratory nature of these analyses, this finding should be interpreted cautiously and confirmed in larger prospective studies.

A central methodological decision in our protocol was the use of non-activated LP-PRP, with platelet activation allowed to occur through endogenous tissue signals. This approach was based on the hypothesis, supported by regenerative medicine studies, that tissue-mediated activation may provide a more physiological release profile of platelet-derived growth factors than exogenous activation with calcium chloride or thrombin [8,9,11]. The mechanisms responsible for platelet activation in vivo remain incompletely understood. Tendons and entheses contain a complex environment composed of collagen fibers, extracellular matrix components, mechanical stress gradients, and inflammatory mediators, all of which may contribute to platelet activation [10]. Avoiding exogenous activation may therefore allow platelet degranulation to occur in closer interaction with the local tissue microenvironment.

Comparative in vitro studies have also suggested that non-activated PRP may provide a more gradual and sustained release of growth factors. In contrast, exogenous activation may produce a rapid initial release followed by an earlier decline [12]. Whether these biological differences translate into clinically meaningful advantages in inflammatory tendon or entheseal disease remains uncertain. Nevertheless, the clinical and imaging improvements observed in the present cohorts indicate that a non-activated LP-PRP strategy is feasible and potentially beneficial in this setting.

Standardization of PRP products remains a major challenge when interpreting the literature. Our protocol used an LP-PRP preparation characterized according to the DEPA classification proposed by Magalon et al., which provides a standardized framework for characterizing platelet dose, production efficiency, purity, and activation [26]. Particular attention was paid to minimizing contamination by red blood cells and leukocytes, as these cellular components may contribute to undesired inflammatory responses.

This approach aligns with recommendations from recent rheumatologic reviews emphasizing the importance of accurately characterizing PRP formulations in clinical studies [20,21]. The consistency of the clinical outcomes observed across the two participating centers further supports the importance of standardized PRP preparation and ultrasound-guided administration when evaluating biological treatments.

Overall, both cohorts demonstrated substantial improvements during the three-month follow-up, including marked pain reduction and progressive improvement in ultrasound abnormalities. In the plantar fascia cohort, PWD activity disappeared by the second month, and MRI-detected BME was absent at the three-month assessment. In the RA cohort, effusion and PWD activity progressively resolved, while partial tendon tears were no longer detectable at three months. Because these imaging outcomes were analyzed descriptively, their evolution should be interpreted as an observed longitudinal pattern rather than as evidence of statistically demonstrated treatment effects.

The disappearance of an imaging abnormality at follow-up indicates that the finding was no longer detectable using the imaging method employed; it should not be interpreted as histological proof of complete tissue regeneration. This distinction is particularly relevant for categorical findings such as Doppler activity, effusion, BME, and partial tendon tears.

Systemic therapies remained unchanged throughout the observational period. This reduces the likelihood that the observed short-term changes were related to modifications in systemic disease-modifying treatment. Nevertheless, without a control group, the observed improvements cannot be definitively attributed to PRP, and spontaneous variation, regression to the mean, concomitant conservative measures, and other unmeasured factors cannot be excluded.

Strengths and limitations

This study has several strengths. It includes a clinically relevant cohort of patients with inflammatory arthritis and foot and ankle involvement evaluated across two expert centers using a standardized LP-PRP preparation and consistent ultrasound-guided techniques. Clinical data were collected prospectively during routine care, and systematic imaging assessment allowed longitudinal evaluation of both symptomatic and structural changes. Systemic antirheumatic therapies remained unchanged, reducing one important potential source of confounding during follow-up.

Several limitations should also be acknowledged. First, the observational design and absence of a control or placebo group preclude definitive causal conclusions regarding PRP efficacy. Second, the cohorts were heterogeneous with respect to disease activity and structural abnormalities, and subgroup analyses had limited statistical power. Third, follow-up was restricted to three months, preventing assessment of long-term durability, recurrence, or delayed adverse events. The study did not include functional or patient-reported outcome measures beyond pain, and neither patients nor evaluators were blinded, introducing the possibility of expectation and measurement bias. Selection bias cannot be excluded, and ultrasound assessment is inherently operator-dependent; no formal interobserver or intraobserver reliability analysis was performed within the routine-care setting. The highly consistent evolution of some imaging findings should therefore be regarded as an observed descriptive pattern requiring confirmation in independent prospective studies. Additionally, several ultrasound outcomes, including PWD activity, effusion, and partial tendon tears, were evaluated descriptively rather than through longitudinal inferential models. Finally, the study was conducted in two highly specialized centers, which may limit the generalizability of the findings to broader clinical settings.

## Conclusions

In this dual-center observational experience, LP-PRP was associated with substantial clinical improvement and progressive changes in imaging abnormalities in patients with plantar fascia involvement related to SpA/PsA and tibialis posterior tendon pathology in RA. Treatment was well tolerated, and no disease flares were observed during the three-month follow-up, including among patients with active inflammatory disease at baseline.

These findings provide preliminary support for standardized, ultrasound-guided LP-PRP as a potential adjunctive treatment for refractory tendon and entheseal manifestations in inflammatory rheumatic diseases. The observed improvements occurred without changes in systemic antirheumatic therapy, although the absence of a control group precludes definitive attribution of these effects to PRP. Further prospective randomized controlled studies with longer follow-up are needed to confirm efficacy and safety and to determine how PRP composition, activation strategies, and injection protocols influence clinical outcomes.
